# Supplementary material for: Versatile and efficient genome editing with Neisseria cinerea Cas9
Source: Commun Biol. 2022 Nov 26;5:1296. doi: 10.1038/s42003-022-04258-z (PMC9701194; doi:10.1038/s42003-022-04258-z)
Supplement: Supplementary file 2 — Supplementary information [file 42003_2022_4258_MOESM2_ESM.pdf]

Supplementary Information

Versatile and efficient genome editing with *Neisseria cinerea* Cas9

Zhiquan Liu[1#], Siyu Chen[1#], Wanhua xie[2], Hao Yu[1], Liangxue Lai[1,3,4,5*], Zhanjun Li[1*]

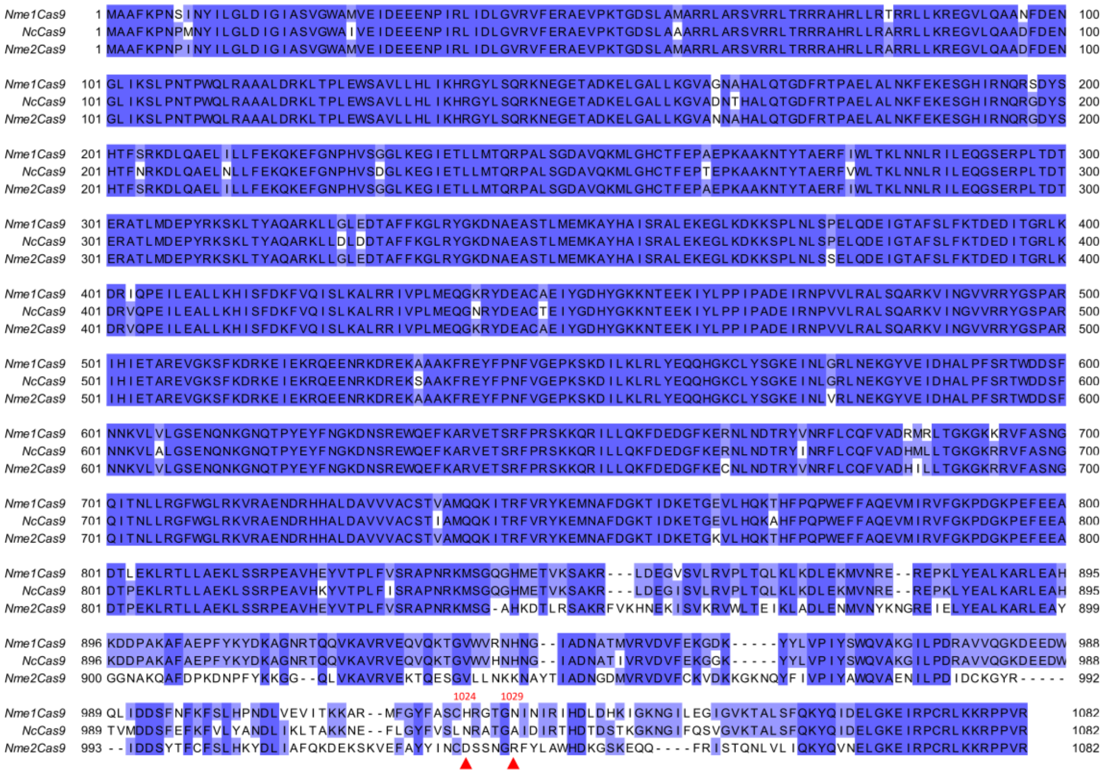

Figure S1. Amino acid sequence alignment of Nme1Cas9, Nme2Cas9 and NcCas9 orthologues. Alignment was performed with Clustal Omega[1] and rendered using Jalview[2]. The PAM-interacting H1024 and N1029 residues are indicated in red.

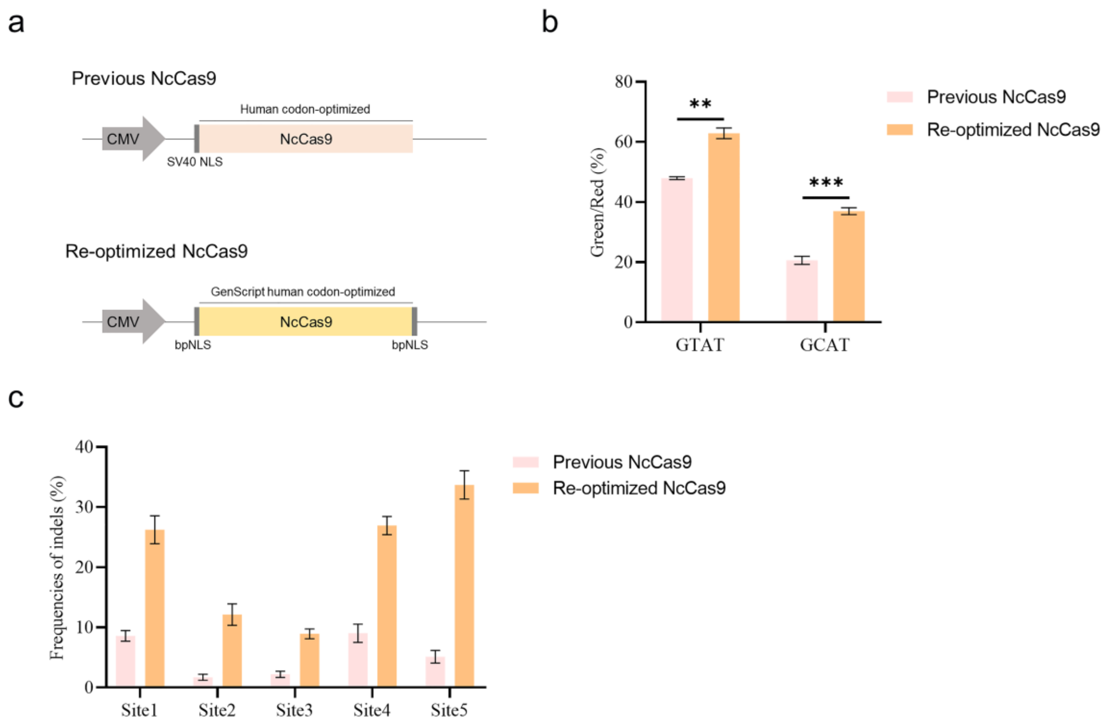

Figure S2. **(a)** Schematic architecture of previously reported NcCas9[3] and our re-optimized NcCas9. **(b)** Comparison of the editing efficiency by pmTmG reporter assays. Red or green represents the tdTomato or EGFP signal, respectively. **(c)** Comparison of the editing efficiency by 5 endogenous loci. Error bars indicate the s.e.m. (n=3).

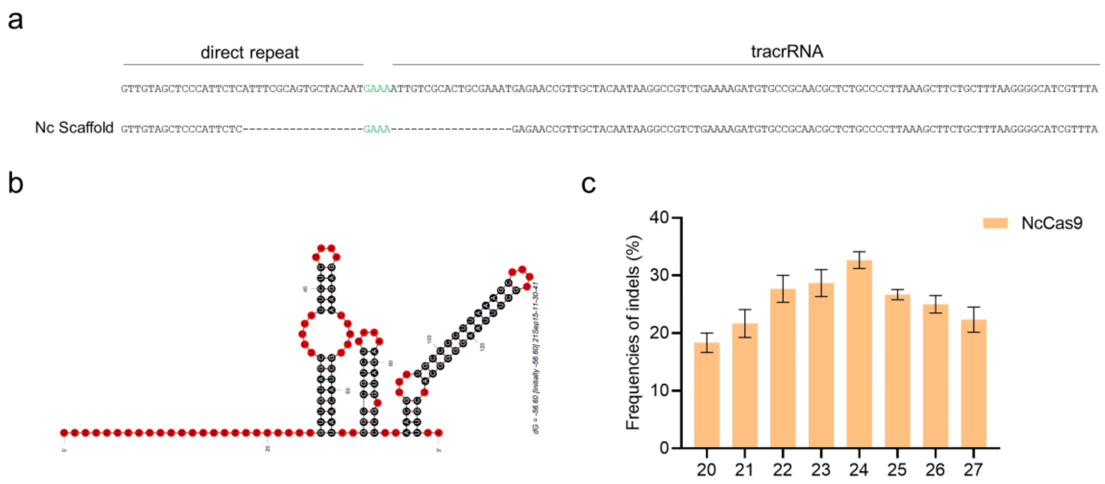

Figure S3. **(a)** Schematic diagram of used Nc sgRNA scaffold in this study. The tetraloop is shown in green. **(b)** The Nc sgRNA structures were obtained using the mfold web server[4]. **(c)** Comparison of NcCas9 editing efficiency of variable spacer lengths in site5. Error bars indicate the s.e.m. (n=3).

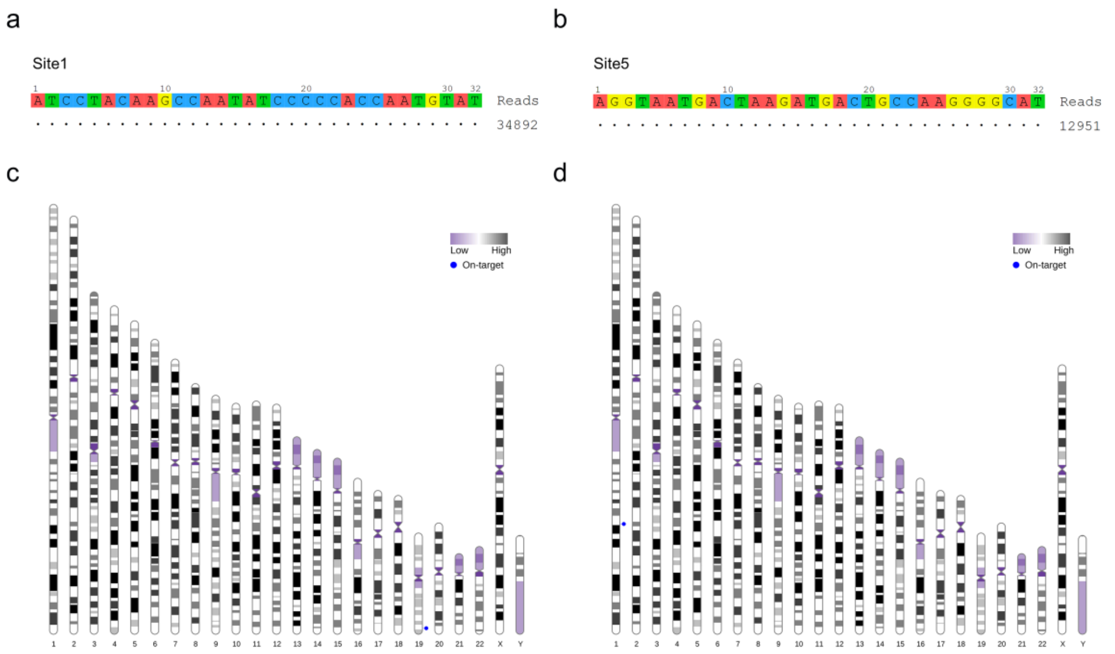

Figure S4. Guide-seq analysis. (**a-b**) On-target and off-target sites evaluated using GUIDE-seq with NcCas9 and sgRNA targeting Site1 (**a**) and Site5 (**b**). (**c-d**) Visual image reveals on-target and off-target sites with dots on chromosomes in Site1 (**c**) and Site5 (**d**).

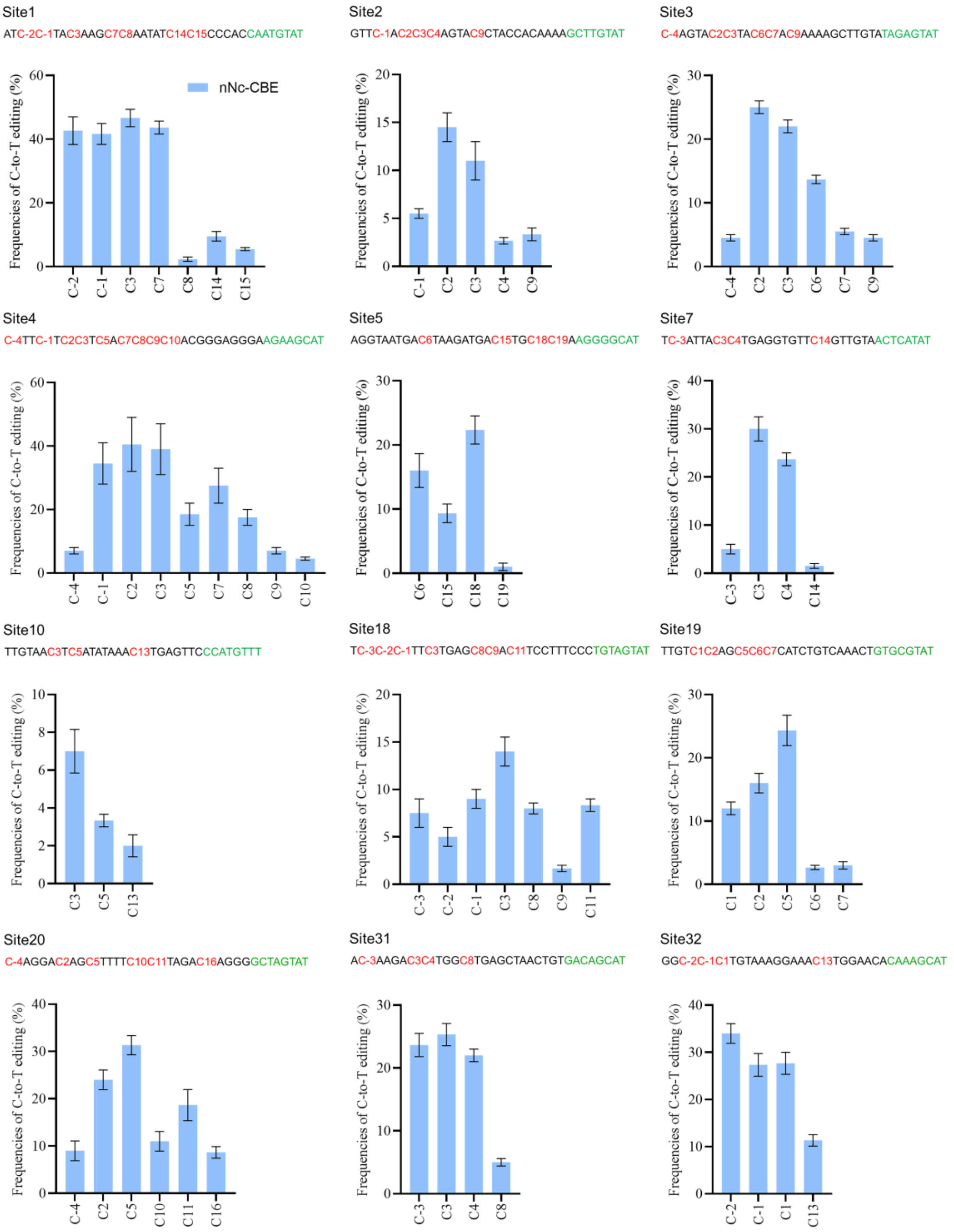

Figure S5. The C-to-T editing frequencies induced by nNc-CBE on each cytosine at 12 target sites. The edited Cs and PAM are shown in red and green, respectively.

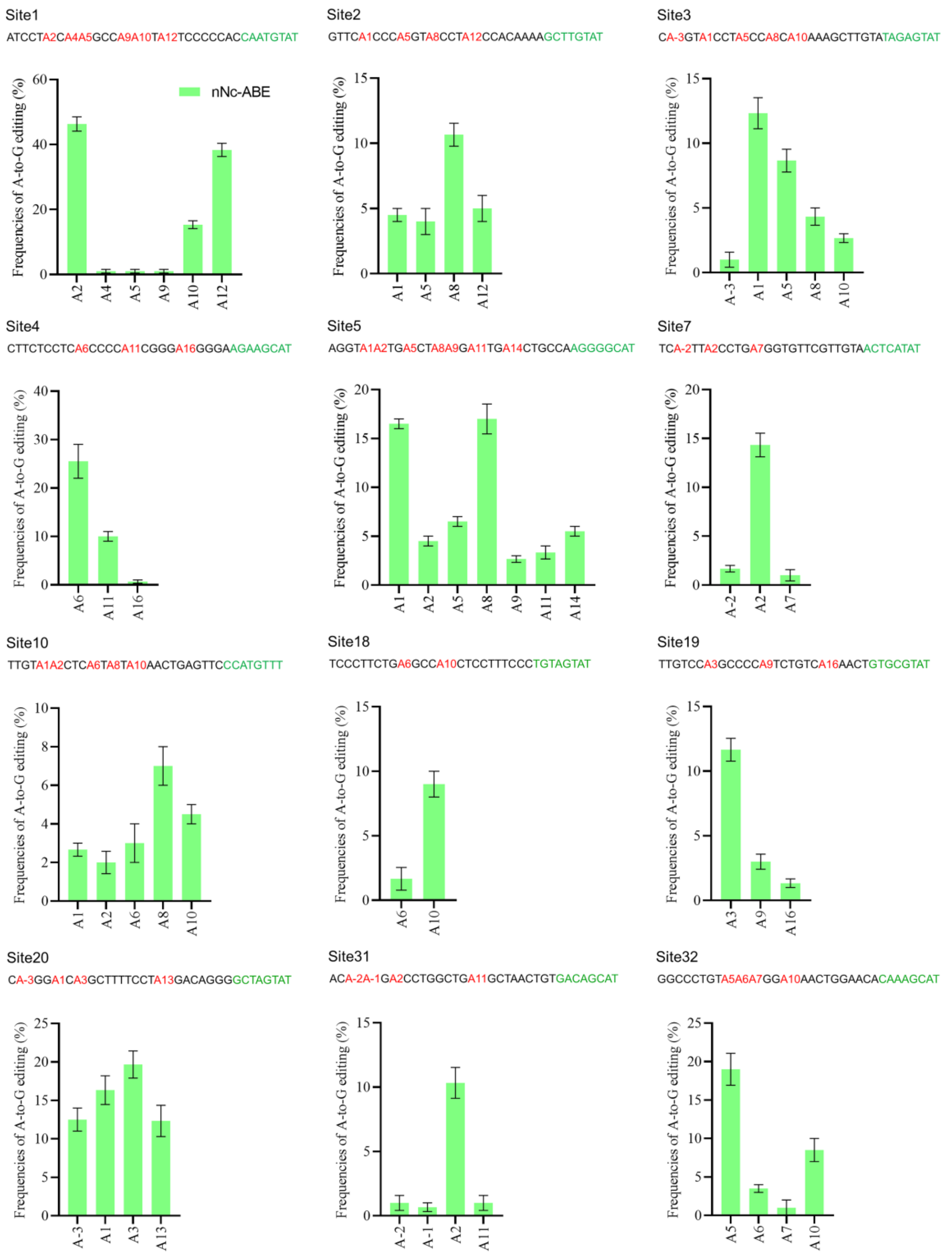

Figure S6. The A-to-G editing frequencies induced by nNc-ABE on each adenine at 12 target sites. The edited As and PAM are shown in red and green, respectively.

Table S1. Primers used for site-directed mutation in this study.

Mutation	Primers (5'-3')
nNcCas9 (D16A)	F: ACATCCTGGGGCTGGCCATCGGAATCGCAA<br>R: GCCAGCCCCAGGATGTAGTTCATAGGGTTT

Table S2. Target sites used in this study. Target sequence (black), PAM region (green).

PAM	Target site	Locus	Sequence (5'-3')
N4GTAT	Site1	*AAVS1*	ATCCTACAAGCCAATATCCCCCACCAATGTAT
	Site2	*RNF2*	GTTCACCCAGTACCTACCACAAAAGCTTGTAT
	Site3	*RNF2*	CAGTACCTACCACAAAAGCTTGTATAGAGTAT
	Site13	*AAVS1*	CCAGGGGGTCTGTGAACTTTCTGACGTTGTAT
	Site14	*RNF2*	AATTTATATTCAAGCATTCCTGACTTCTGTAT
	Site15	*CHM13 chromosome 4*	TTAATAGATACTTATGAACACTAGCCTTGTAT
	Site16	*CHM13 chromosome 4*	ACTTACCTTATCCTGGAGTCTATGCTTAGTAT
	Site17	*CHM13 chromosome 4*	GAGACACATACAAGGCTAGTGTTCATAAGTAT
	Site18	*CHM13 chromosome 4*	TCCCTTCTGAGCCACTCCTTTCCCTGTAGTAT
	Site19	*VISTA hs267*	TTGTCCAGCCCCATCTGTCAAACTGTGCGTAT
	Site20	*LINC01509*	CAGGACAGCTTTTCCTAGACAGGGGCTAGTAT
	Site21	*LINC01509*	CTTCCAGCCCAGCCAAACTTGTCAACCAGTAT
	Site22	*DYRK1A*	CCTCTAACTAACCAGGTAAGTTCATGGAGTAT
	Site23	*DYRK1A*	ACTCAACACAACAGTTATGTTTTTAATGGTAT
	Site24	*DYRK1A*	TAAAGGAAATATTCATATCCTAATGTTTGTAT
	Site25	*POR*	CTGGTGCACCCCAGGCTCAGTCTGCCGTGTAT
N4GCAT	Site4	*AAVS1*	CTTCTCCTCACCCCACGGGAGGGAAGAAGCAT
	Site5	*RNF2*	AGGTAATGACTAAGATGACTGCCAAGGGGCAT
	Site6	*FANCF*	GGGAGATTGACATGCATTTCGACCAATAGCAT
	Site26	*FANCF*	TGCGCCACATCCATCGGCGCTTTGGTCGGCAT
	Site27	*EMX1*	AAGGGCTCCCATCACATCAACCGGTGGCGCAT
	Site28	*CHM13 chromosome 4*	AATGAAGTTATGATCGAAAGAATACTAAGCAT
	Site29	*CHM13 chromosome 4*	AACACTAGCCTTGTATGTGTCTCAGGTGGCAT
	Site30	*CHM13 chromosome 4*	CAGCTTCCTCGGACTTTGGGGTAGGTTTGCAT
	Site31	*VISTA hs267*	ACAAGACCTGGCTGAGCTAACTGTGACAGCAT
	Site32	*VISTA hs267*	GGCCCTGTAAAGGAAACTGGAACACAAAGCAT
	Site33	*LINC01509*	AGGCCAGGGCTGGAGAAGCAGAAAAAAAGCAT
	Site34	*LINC01509*	ACCCAGAGCCCAGTGGCTTTCAGCACCTGCAT
	Site35	*DYRK1A*	TTATCTTCTTGTAGGAGGAGAGACTTCAGCAT
	Site36	*DYRK1A*	ACGGTGCAAGCCGAACAGATGAAGGTTTGCAT
	Site37	*POR*	CGGCCTCACCCTTGGTCTCCCCTTTCCAGCAT
	Site38	*POR*	GCCCATCTCCCCCATGTACACCTTGGCCGCAT
N4ATAT	Site7	*RNF2*	TCATTACCTGAGGTGTTCGTTGTAACTCATAT
	Site8	*RNF2*	CCAACATACAGAAGTCAGGAATGCTTGAATAT
	Site9	*RNF2*	TAAAAATGTATCCCAGTTTACACGTCTCATAT
	Site39	*CHM13 chromosome 4*	AAGCATAGACTCCAGGATAAGGTAAGTAATAT
	Site40	*CHM13 chromosome 4*	TTGTATGTGTCTCAGGTGGCATGAGGCAATAT
	Site41	*VISTA hs267*	AGTACCACCAGTTTTATGAAGAAAAATGATAT
	Site42	*DYRK1A*	CTAAAGTTCTTATTTAAGGAACCATTAGATAT
	Site43	*DYRK1A*	AGATATGTCAAATGATACAAACATTAGGATAT

N4GTTT	Site10	*RNF2*	TTGTAACTCATATAAACTGAGTTCCCATGTTT
	Site11	*RNF2*	ATTTATTATTACTCTATGTTCTATTTAAGTTT
	Site12	*RNF2*	AGTTTTCATGTTCTAAAAATGTATCCCAGTTT
	Site44	*EMX1*	TCAGGCCCTTCCTCCTCCAGCTTCTGCCGTTT
	Site45	*EMX1*	AGCAGCAAGCAGCACTCTGCCCTCGTGGGTTT
	Site46	*DYRK1A*	TAACTGTTGTGTTGAGTAACATATACCTGTTT
	Site47	*DYRK1A*	TAACATATACCTGTTTGTAGTTAGAAAAGTTT
	Site48	*DYRK1A*	TCGCCAGCCAAACATAAGTGACCAACAGGTTT
N4GYAT	Tyr-1	*Tyr*	TAATGATATCAACATCTACGACCTCTTTGTAT
	Tyr-2	*Tyr*	TCAACATCTACGACCTCTTTGTATGGATGCAT
	Pcsk9-1	*Pcsk9*	GGGAAGATGGAAGCAGCCAGGTGGAGGTGTAT
	Pcsk9-2	*Pcsk9*	GAGGAAGACTCCTTTGTCTTCGCCCAGAGCAT

Table S3. Primers used for genotyping in this study.

Primers	Sequence (5'-3')	Product size (bp)	Target sites
AAVS1-F:<br>AAVS1-R:	GCAGTCGCCTTGTGATTTG<br>GCGTTTCTCCTTGACATTCTTT	391	Site1, 4, 13
RNF2-F:<br>RNF2-R:	GTCTTCCTTGGTGCCTTATCA<br>TGGTGGGACACATACATTCAG	524	Site2, 3, 5, 7-12, 14
FANCF-F:<br>FANCF-R:	ATCTGCTCTCCCTCCACTAA<br>GGCCTGGAAGTTCGCTAAT	483	Site6
CHM13-F:<br>CHM13-R:	CATCTTCTCCGCAGACAGAAATA<br>TCCTGCCCTTAGGTCAAATAAC	465	Site15-18, 28-30, 39, 40
VISTA-F:<br>VISTA-R:	GGACGTCTGCCCAATATGTAA<br>GCATTAATCCACAGCAACACC	455	Site19, 31, 32, 41
LINC-F:<br>LINC-R:	GAAACGCCCATGCAATTAGTC<br>ATCACTTTCTGGCCTCAGTTT	443	Site20, 21, 33, 34
DYRK1A-F:<br>DYRK1A-R:	GGAGCTGGTCTGTTGGAGAA<br>TCCCAATCCATAATCCCACGTT	692	Site22-24, 35, 36, 42, 43, 46-48
POR-F:<br>POR-R:	CTCCCCTGCTTCTTGTCGTAT<br>ACAGGTCGTGGACACTCACA	380	Site 25, 37, 38
EMX1-F:<br>EMX1-R:	CCTTCTGTGAATGTTAGACCCA<br>TGCTTGTCCCTCTGTCAATG	520	Site27, 44, 45
Tyr-F:<br>Tyr-R:	CAGTGCTCAGGCAACTTCAT<br>CGACCTCCCAAGTACTCATCT	497	Tyr-1, Tyr-2
Pcsk9-F1:<br>Pcsk9-R1:	CCCACTCCTTGCTGTAAGATT<br>CACACTTGCTCGCCTGAT	445	Pcsk9-1
Pcsk9-F2:<br>Pcsk9-R2:	CCCAGCCAGAGAGAAATGAG<br>GACTAAGACAGGAAGGGATGAAG	489	Pcsk9-2

Supplementary Note 1

Within the sequences below, NcCas9 sequence is in gray and bpNLS sequences are in green.

Amino acid sequence of NcCas9.

MKRTADGSEFESPKKKRKVAAFKPNPMNYILGLDIGIASVGWAIVEIDEEENPIRLIDLGVR
VFERAEVPKTGDSLAAARRLARSVRRLTRRRAHRLLRARRLLKREGVLQAADFDENGLI
KSLPNTPWQLRAAALDRKLTPLEWSAVLLHLIKHRGYLSQRKNEGETADKELGALLKGV
ADNTHALQTGDFRTPAELALNKFEKESGHIRNQRGDYSHTFNRKDLQAELNLLFEKQKEF
GNPHVSDGLKEGIETLLMTQRPALSGDAVQKMLGHCTFEPTEPKAAKNTYTAERFVWLT
KLNNLRILEQGSERPLTDTERATLMDEPYRKSKLTYAQARKLLDLDDTAFFKGLRYGKDN
AEASTLMEMKAYHAISRALEKEGLKDKKSPLNLSPELQDEIGTAFSLFKTDEDITGRLKDR
VQPEILEALLKHISFDKFVQISLKALRRIVPLMEQGNRYDEACTEIYGDHYGKKNTEEKIY
LPPIPADEIRNPVVLRALSQARKVINGVVRRYGSPARIHIETAREVGKSFKDRKEIEKRQEE
NRKDREKSAAKFREYFPNFVGEPKSKDILKLRLYEQQHGKCLYSGKEINLGRLNEKGYVE
IDHALPFSRTWDDSFNNKVLALGSENQNKGNQTPYEYFNGKDNSREWQEFKARVETSRF
PRSKKQRILLQKFDEDGFKERNLNDTRYINRFLCQFVADHMLLTGKGKRRVFASNGQITNL
LRGFWGLRKVRAENDRHHALDAVVVACSTIAMQQKITRFVRYKEMNAFDGKTIDKETGE
VLHQKAHFPQPWEFFAQEVMIRVFGKPDGKPEFEEADTPEKLRTLLAEKLSSRPEAVHKY
VTPLFISRAPNRKMSGQGHMETVKSAKRLDEGISVLRVPLTQLKLKDLEKMVNREREPKL
YEALKARLEAHKDDPAKAFAEPFYKYDKAGNRTQQVKAVRVEQVQKTGVWVHNHNGI
ADNATIVRVDVFEKGGKYYLVPIYSWQVAKGILPDRAVVQGKDEEDWTVMDDSFEFKFV
LYANDLIKLTAKKNEFLGYFVSLNRATGAIDIRTHDTDSTKGKNGIFQSVGVKTALSFQKY
QIDELGKEIRPCRLKKRPPVRSGGSKRTADGSEFEPKKKRKV

DNA sequence of NcCas9.

atgaaacggacagccgacggaagcgagttcgagtcaccaaagaagaagcggaaagtcGCCGCCTTCAAACCAAACCC
TATGAACTACATCCTGGGGCTGGACATCGGAATCGCAAGCGTGGGCTGGGCTATCGTG
GAAATCGACGAGGAGGAGAACCCAATCCGGCTGATCGACCTGGGCGTGAGAGTGTTT
GAGAGGGCAGAGGTGCCAAAGACCGGCGATTCCCTGGCAGCAGCACGGAGACTGGC
ACGGTCTGTGAGGCGCCTGACACGGAGAAGGGCACACAGACTGCTGAGGGCACGCC
GGCTGCTGAAGAGAGAGGGCGTGCTGCAGGCAGCAGACTTCGATGAGAACGGCCTGA
TCAAGAGCCTGCCTAATACCCCATGGCAGCTGAGAGCAGCCGCCCTGGACAGGAAGC
TGACACCACTGGAGTGGTCCGCCGTGCTGCTGCACCTGATCAAGCACCGCGGCTACCT
GTCTCAGCGGAAGAATGAGGGCGAGACAGCCGATAAGGAGCTGGGCGCCCTGCTGAA
GGGAGTGGCCGACAACACACACGCACTGCAGACCGGCGACTTCCGGACACCTGCAGA
GCTGGCCCTGAATAAGTTCGAGAAGGAGAGCGGACACATCAGGAACCAGAGGGGCGA
CTATTCCCACACCTTCAACCGCAAGGATCTGCAGGCCGAGCTGAATCTGCTGTTTGAG
AAGCAGAAGGAGTTCGGCAATCCTCACGTGTCCGACGGCCTGAAGGAGGGCATCGAG
ACACTGCTGATGACCCAGAGGCCAGCCCTGTCTGGCGATGCAGTGCAGAAGATGCTG
GGCCACTGCACATTTGAGCCCACCGAGCCTAAGGCCGCCAAGAACACCTACACAGCC
GAGAGGTTCGTGTGGCTGACAAAGCTGAACAATCTGAGGATCCTGGAGCAGGGAAGC
GAGAGGCCTCTGACCGACACAGAGAGGGCCACCCTGATGGATGAGCCATACAGGAAG

TCCAAGCTGACATATGCACAGGCAAGGAAGCTGCTGGACCTGGACGATACCGCCTTCT
TTAAGGGCCTGAGATACGGCAAGGATAATGCCGAGGCCTCTACACTGATGGAGATGAA
GGCCTATCACGCCATCAGCCGCGCCCTGGAGAAGGAGGGCCTGAAGGACAAGAAGTC
TCCTCTGAACCTGAGCCCAGAGCTGCAGGATGAGATCGGCACCGCCTTTTCCCTGTTC
AAGACCGACGAGGACATCACAGGCCGCCTGAAGGACAGGGTGCAGCCTGAGATCCTG
GAGGCCCTGCTGAAGCACATCTCTTTTGATAAGTTCGTGCAGATCAGCCTGAAGGCCC
TGAGAAGGATCGTGCCACTGATGGAGCAGGGCAATCGCTACGACGAGGCCTGCACCG
AGATCTACGGCGATCACTATGGCAAGAAGAACACAGAGGAGAAGATCTATCTGCCCCC
TATCCCAGCCGACGAGATCAGAAATCCAGTGGTGCTGAGGGCCCTGAGCCAGGCACG
CAAAGTGATCAACGGAGTGGTGCGCCGGTACGGCTCCCCCGCAAGGATCCACATCGA
GACAGCCAGAGAAGTGGGCAAGTCTTTTAAGGACCGGAAGGAGATCGAGAAGAGAC
AGGAGGAGAATAGAAAGGATAGGGAGAAGAGCGCCGCCAAGTTCAGGGAGTACTTTC
CCAACTTCGTGGGCGAGCCTAAGTCCAAGGACATCCTGAAGCTGCGCCTGTACGAGCA
GCAGCACGGCAAGTGTCTGTATAGCGGCAAGGAGATCAACCTGGGCCGGCTGAATGA
GAAGGGCTATGTGGAGATCGATCACGCCCTGCCCTTTAGCAGAACCTGGGACGATTCC
TTCAACAATAAGGTGCTGGCCCTGGGCTCTGAGAACCAGAATAAGGGCAATCAGACAC
CATACGAGTATTTCAACGGCAAGGACAATAGCAGGGAGTGGCAGGAGTTTAAGGCAA
GGGTGGAGACAAGCAGGTTCCCACGGTCTAAGAAGCAGCGGATCCTGCTGCAGAAGT
TTGACGAGGATGGCTTCAAGGAGAGAAACCTGAATGACACCAGATACATCAACAGGTT
TCTGTGCCAGTTCGTGGCCGATCACATGCTGCTGACCGGCAAGGGCAAGAGAAGGGT
GTTTGCCTCCAACGGCCAGATCACAAATCTGCTGAGGGGCTTCTGGGGCCTGAGAAAG
GTGAGGGCAGAGAATGACAGGCACCACGCACTGGATGCAGTGGTGGTGGCATGTAGC
ACCATCGCCATGCAGCAGAAGATCACACGCTTTGTGCGGTATAAGGAGATGAACGCCT
TCGACGGCAAGACCATCGATAAGGAGACAGGCGAGGTGCTGCACCAGAAGGCCCACT
TTCCACAGCCCTGGGAGTTCTTTGCCCAGGAAGTGATGATCCGGGTGTTTGGCAAGCC
AGACGGCAAGCCCGAGTTCGAGGAGGCCGATACCCCAGAGAAGCTGCGGACACTGCT
GGCAGAGAAGCTGAGCTCCAGACCTGAGGCCGTGCACAAGTACGTGACCCCTCTGTT
CATCTCCCGCGCCCCAAATCGGAAGATGTCTGGCCAGGGCCACATGGAGACAGTGAA
GTCTGCCAAGCGGCTGGACGAGGGCATCAGCGTGCTGAGAGTGCCCCTGACACAGCT
GAAGCTGAAGGATCTGGAGAAGATGGTGAACCGCGAGCGGGAGCCTAAGCTGTATGA
GGCCCTGAAGGCCAGACTGGAGGCCCACAAGGACGATCCCGCCAAGGCCTTTGCCGA
GCCTTTCTACAAGTATGACAAGGCCGGCAATCGGACCCAGCAGGTGAAGGCCGTGAG
AGTGGAGCAGGTGCAGAAGACAGGCGTGTGGGTGCACAACCACAATGGCATCGCCGA
CAACGCCACAATCGTGCGGGTGGACGTGTTCGAGAAGGGCGGCAAGTACTATCTGGT
GCCAATCTACTCCTGGCAGGTGGCAAAGGGAATCCTGCCAGACAGAGCCGTGGTGCA
GGGCAAGGACGAGGAGGATTGGACCGTGATGGACGATTCTTTCGAGTTTAAGTTCGTG
CTGTACGCCAACGACCTGATCAAGCTGACCGCCAAGAAGAATGAGTTTCTGGGCTATT
TCGTGTCTCTGAACAGGGCAACAGGAGCAATCGACATCAGGACCCACGACACAGATA
GCACCAAGGGCAAGAACGGCATCTTTCAGAGCGTGGGCGTGAAGACCGCCCTGTCCT
TCCAGAAGTATCAGATTGACGAACTGGGAAAAGAGATTAGACCCTGTAGGCTGAAAA
AGAGACCACCTGTGAGAtctggcggctcaaaagaaccgccgacggcagcgaattcgagcccaagaagaagaggaaagt
c

Supplementary references

1. Sievers F*, et al.* Fast, scalable generation of high-quality protein multiple sequence alignments using Clustal Omega. *Molecular systems biology* **7**, 539 (2011).

2. Waterhouse AM, Procter JB, Martin DM, Clamp M, Barton GJ. Jalview Version 2--a multiple sequence alignment editor and analysis workbench. *Bioinformatics (Oxford, England)* **25**, 1189-1191 (2009).

3. Ran FA*, et al.* In vivo genome editing using Staphylococcus aureus Cas9. *Nature* **520**, 186-191 (2015).

4. Zuker M. Mfold web server for nucleic acid folding and hybridization prediction. *Nucleic acids research* **31**, 3406-3415 (2003).
