# Supplementary material for: Versatile and efficient genome editing with Neisseria cinerea Cas9
Source: Commun Biol. 2022 Nov 26;5:1296. doi: 10.1038/s42003-022-04258-z (PMC9701194; doi:10.1038/s42003-022-04258-z)
Supplement: Supplementary file 5 — Reporting Summary [file 42003_2022_4258_MOESM5_ESM.pdf]

nature portfolio

Corresponding author(s): Zhanjun Li

Last updated by author(s): Oct 27, 2022

Reporting Summary

Nature Portfolio wishes to improve the reproducibility of the work that we publish. This form provides structure for consistency and transparency in reporting. For further information on Nature Portfolio policies, see our Editorial Policies and the Editorial Policy Checklist.

Statistics

For all statistical analyses, confirm that the following items are present in the figure legend, table legend, main text, or Methods section.

n/a	Confirmed	
☐	☒	The exact sample size (n) for each experimental group/condition, given as a discrete number and unit of measurement
☐	☒	A statement on whether measurements were taken from distinct samples or whether the same sample was measured repeatedly
☐	☒	The statistical test(s) used AND whether they are one- or two-sided<br>*Only common tests should be described solely by name; describe more complex techniques in the Methods section.*
☒	☐	A description of all covariates tested
☒	☐	A description of any assumptions or corrections, such as tests of normality and adjustment for multiple comparisons
☒	☐	A full description of the statistical parameters including central tendency (e.g. means) or other basic estimates (e.g. regression coefficient) AND variation (e.g. standard deviation) or associated estimates of uncertainty (e.g. confidence intervals)
☒	☐	For null hypothesis testing, the test statistic (e.g. F, t, r) with confidence intervals, effect sizes, degrees of freedom and P value noted<br>*Give P values as exact values whenever suitable.*
☒	☐	For Bayesian analysis, information on the choice of priors and Markov chain Monte Carlo settings
☒	☐	For hierarchical and complex designs, identification of the appropriate level for tests and full reporting of outcomes
☒	☐	Estimates of effect sizes (e.g. Cohen's d, Pearson's r), indicating how they were calculated

Our web collection on statistics for biologists contains articles on many of the points above.

Software and code

Policy information about availability of computer code

Data collection	The indels efficiencies and base editing efficiencies were quantified by TIDE and EditR 1.0.10, respectively.
Data analysis	The data were analysed by two-sided t test via GraphPad Prism software 8.0.1. A probability value smaller than 0.05 (p < 0.05) was considered to be statistically significant. *p < 0.05, **p < 0.01, ***p < 0.001.

For manuscripts utilizing custom algorithms or software that are central to the research but not yet described in published literature, software must be made available to editors and reviewers. We strongly encourage code deposition in a community repository (e.g. GitHub). See the Nature Portfolio guidelines for submitting code & software for further information.

Data

Policy information about availability of data

All manuscripts must include a data availability statement. This statement should provide the following information, where applicable:

- Accession codes, unique identifiers, or web links for publicly available datasets
- A description of any restrictions on data availability
- For clinical datasets or third party data, please ensure that the statement adheres to our policy

All data generated or analysed during this study are included in this published article and its supplementary files.

Field-specific reporting

Please select the one below that is the best fit for your research. If you are not sure, read the appropriate sections before making your selection.

☒ Life sciences ☐ Behavioural & social sciences ☐ Ecological, evolutionary & environmental sciences

For a reference copy of the document with all sections, see nature.com/documents/nr-reporting-summary-flat.pdf

Life sciences study design

All studies must disclose on these points even when the disclosure is negative.

Sample size	The experiments were repeated three independent biological replicates. These sample sizes were selected based on literature that used similar sample sizes to obtain gene editing results.
Data exclusions	No data weas excluded.
Replication	Values and error bars reflect the mean ± s.e.m. of three independent biological replicates.
Randomization	Due to the small sample, randomization was not applicable for this study.
Blinding	Blinding was not relevant to our study. In general, based on the prior experience of other groups in the field, these types of assays do not require blinding.

Reporting for specific materials, systems and methods

We require information from authors about some types of materials, experimental systems and methods used in many studies. Here, indicate whether each material, system or method listed is relevant to your study. If you are not sure if a list item applies to your research, read the appropriate section before selecting a response.

Materials & experimental systems

n/a	Involved in the study	
☒	☐	Antibodies
☐	☒	Eukaryotic cell lines
☒	☐	Palaeontology and archaeology
☐	☒	Animals and other organisms
☒	☐	Human research participants
☒	☐	Clinical data
☒	☐	Dual use research of concern

Methods

n/a	Involved in the study	
☒	☐	ChIP-seq
☐	☒	Flow cytometry
☒	☐	MRI-based neuroimaging

Eukaryotic cell lines

Policy information about cell lines

Cell line source(s)	HEK293T (ATCC)
Authentication	The cell lines were authenticated by supplier and not further authenticated after receipt.
Mycoplasma contamination	All cell lines tested negative for mycoplasma.
Commonly misidentified lines (See ICLAC register)	No commonly misidentified cell lines were used in this study.

Animals and other organisms

Policy information about studies involving animals; ARRIVE guidelines recommended for reporting animal research

Laboratory animals	C57/BL6 mice. Mice were maintained in an Assessment and Accreditation of Laboratory Animal Care credited specific pathogen free facility under a 12 h dark-light cycle. Ambient temperature is 20 centigrade, relative humidity is 50%.
Wild animals	This study did not involve wild animals.
Field-collected samples	This study did not involve field-collected samples.

Ethics oversight | All animal studies were conducted under the guidance of the Animal Welfare and Research Ethics Committee at Jilin University.

Note that full information on the approval of the study protocol must also be provided in the manuscript.

Flow Cytometry

Plots

Confirm that:

- [x] The axis labels state the marker and fluorochrome used (e.g. CD4-FITC).
- [x] The axis scales are clearly visible. Include numbers along axes only for bottom left plot of group (a 'group' is an analysis of identical markers).
- [x] All plots are contour plots with outliers or pseudocolor plots.
- [x] A numerical value for number of cells or percentage (with statistics) is provided.

Methodology

Sample preparation	HEK293T cells were digested by trypsin, centrifuged at 1000 rpm and filtered with a 35 um nylon mesh.
Instrument	BD FACSAriaII
Software	FlowJo_V10
Cell population abundance	HEK293T cells were co-transfected with Nme1Cas9/Nme2Cas9/NcCas9, corresponding sgRNA, SaCas9 and its sgRNA, and pmTmG library plasmids (1:1:1:1:1).Cells were harvested for editing quantification by flow cytometry. Quantification was based on the relative fluorescent frequencies of green/red.
Gating strategy	Positive and negative boundaries were determined by control cells that were not transfected with any plasmids.

- [ ] Tick this box to confirm that a figure exemplifying the gating strategy is provided in the Supplementary Information.
